# Unveiling the Identity of *Unio crassus* (Bivalvia: Unionidae) in Central and Eastern Europe

**DOI:** 10.3390/genes17101282

**Published:** 2026-10-14

**Authors:** Agnieszka Kaczmarczyk-Ziemba, Jerzy Sell, Katarzyna Zając, Tadeusz Zając, Adrianna Kilikowska, Anna Wysocka, Marcin Górniak, Monika Mioduchowska

**Affiliations:** 1 Department of Evolutionary Genetics and Biosystematics, Faculty of Biology, University of Gdansk, Wita Stwosza 59, 80-308 Gdansk, Poland; jerzy.sell@ug.edu.pl (J.S.); adrianna.kilikowska@gdansk.merito.pl (A.K.); marcin.gorniak@ug.edu.pl (M.G.); 2 Department of Biodiversity, Institute of Nature Conservation, Polish Academy of Sciences, Adama Mickiewicza 33, 31-120 Cracow, Poland; kzajac@iop.krakow.pl; 3 Department of Ecosystem Conservation, Institute of Nature Conservation, Polish Academy of Sciences, Adama Mickiewicza 33, 31-120 Cracow, Poland; tzajac@iop.krakow.pl; 4 Department of Health, WSB Merito University, Grunwaldzka 238A, 80-266 Gdansk, Poland; anna.wysocka@gdansk.merito.pl

**Keywords:** *cox1*, *nadh3*-*tRNA*-*nadh2*, *Unio crassus*, Unionidae, phylogenetic relationships

## Abstract

**Background/Objectives:** Freshwater mussels (Bivalvia: Unionida) are among the most endangered faunal groups. This study examines the genetic diversity of *Unio crassus*, a species critically endangered in Europe. **Methods:** Using novel (*nadh3*-*tRNA*-*nadh2*) and existing (*cox1*) mitochondrial markers, we identified four genetic lineages distributed across Central and Southern Europe. The genetic analysis aligned with a recent integrative approach that recognized *U. crassus* as a complex of 12 geographically structured species. **Results:** Genetic clusters identified in the present study are grouped within *U. crassus* s. str., *U. nanus*, and *U. bruguierianus* clades. Moreover, our results support stronger internal differentiation within *U. nanus* and *U. bruguierianus* than within *U. crassus* s. str. Further analyses confirmed the widespread and overlapping distribution ranges of both *U. crassus* s. str. and *U. nanus*. Moreover, mitochondrial data are consistent with an expanded distribution of the mitochondrial lineage corresponding to *U. bruguierianus* and support its expanded range covering also the Crimean Peninsula and western part of Ukraine. **Conclusions:** Results of time-calibrated phylogenetic analyses conducted for identified clusters were in line with those derived from advanced analyses based on mitogenome sequencing. We showed that selected mitochondrial markers are sufficient to make computations of divergence times within the *U. crassus* complex. The present study suggests that historical climatic events and glacial refugia played a significant role in the divergence within selected freshwater mussels. The findings highlight the importance of genetic analyses in revealing cryptic diversity within threatened species and managing their conservation. Data are deposited in GenBank (accession numbers KT598034–KT598185).

## 1. Introduction

Freshwater mussels (Bivalvia: Unionida) are among the most endangered groups of animals. Currently, the global conservation status of 511 out of 859 recognized species has been assessed through the International Union for Conservation of Nature (IUCN) [1,2]. Increasing anthropogenic pressure resulting in habitat loss, its modification and fragmentation, overexploitation of natural resources, pollution, climate change, as well as the introduction of invasive species can be mentioned among the main threats to mussels’ populations [3,4]. The global decline of freshwater mussels, manifested in reductions in both the number of species and the abundance of populations, as well as a growing number of species facing extinction, is causing increasing concern nowadays [2,5,6,7,8,9,10]. Thus, an insight into genetic diversity has become a principal issue for the conservation and management of Unionida species.

Genetic analyses applied in wide-spatial studies regarding freshwater mussels (especially in a phylogeographic approach) allow identification of separate species within a genus or distinct genetic lineages within species [11,12,13,14,15,16,17]. Those evolutionary lineages can then be managed as separate conservation units to preserve their genetic integrity and evolutionary potential [16], two factors especially important for carefully planned conservation efforts including captive breeding and translocation. Moreover, understanding patterns of genetic variation also makes it possible to reconstruct the phylogenetic and demographic histories of species, as well as their historical distribution changes and migration routes [18,19]. Interestingly, genetic analyses of Unionida species are also extraordinarily useful to understand past geological and hydrological events due to mussels’ stable biogeography connected with their low dispersal abilities and restriction to freshwater habitats [20].

Although studies focused on diversity, distribution and evolution of freshwater mussels are widely supported by complex genetic analyses (e.g., [8,21,22,23,24,25]), the current knowledge is still not without gaps, and open questions often lead to the reevaluation of species status. One of those reevaluations was addressed to the thick-shelled river mussel *Unio crassus* Philipsson, 1788, whose genetic diversity was recently analyzed on a wider spatial scale [14]. *U. crassus* considered the most abundant unionid in Europe, is one of the most threatened with extinction representatives of this group [8]. Over the second half of the last century, the range of *U. crassus* had become fragmented, and the species declined dramatically in Western and Central Europe [26,27,28,29,30,31]. As a result, the thick-shelled river mussel is listed as endangered in the IUCN Red List [8,32,33] and ranked as critically endangered in some European countries, e.g., [34,35,36,37]. Moreover, *U. crassus* holds a high legal conservation status and is a priority target species for freshwater conservation [33]. However, as mentioned above, analyses of genetic variation have not been widely applied in *U. crassus* studies so far. Specimens of the thick-shelled river mussel were included in studies on molecular taxonomy and phylogeny of the *Unio* genus, which gave preliminary insight into its genetic diversity [6,21,37,38,39,40,41,42,43,44,45,46,47]. Complete sequences of both types of mitochondrial genomes (maternal and paternal, respectively) were determined, and comparative analyses of the phylogenies reconstructed using mitogenomic data were performed. Burzyński et al. (2017) [48] determined complete sequences of both types of mitochondrial genomes (maternal and paternal, respectively) and performed comparative analyses of the phylogenies reconstructed using mitogenomic data. Analyses based on maternally and paternally transmitted mitochondrial markers allowed resolution and comparison of phylogenetic relationships among *U. crassus* populations from both perspectives [49,50]. Moreover, analyses of mitochondrial and nuclear (allozyme locus) markers were used to test the hypothesis of reproductive isolation between separate *U. crassus* groups found in Ukraine [51]. However, the knowledge of the *U. crassus* population’s genetic structure is still limited. Polymorphism of microsatellite markers described by Sell et al. (2013) [52] has been analyzed only as a genetic background in studies on host fish compatibility of *U. crassus* [53] and, more recently, in analyses of the genetic structure of the thick-shelled river mussel population on a limited spatial scale [37,54].

More recently, an integrative study shed light on systematics, revealing *U. crassus* to be a complex of 12 geographically structured cryptic species [14]. Two of them (*U. crassus* s. str. and *U. nanus* stat. rev., respectively) are considered to be widely distributed in Europe with overlapping ranges [14,55,56], whereas other delineated species occur mainly in southern regions of the continent. Nevertheless, a proper identification of those species using only shell shape features is hampered by a broad morphospace overlap observed between several species of the complex. Lopes-Lima et al. [14] established an integrative framework for the *U. crassus* complex based on mitochondrial and nuclear data, providing a reference for interpreting the genetic and geographic structure of the complex. The present study does not aim to independently delimit species within the *U. crassus* complex. Instead, we assess whether two mitochondrial markers, *nadh3–tRNA–nadh2* and *cox1*, recover the broad mitochondrial lineage structure corresponding to the units identified in the recent integrative revision. By analyzing specimens collected across Central and Southern Europe and identified as *U. crassus* based on shell morphology, we further expand the geographic sampling of the complex and refine the known distributions of the corresponding mitochondrial lineages. Thus, our study complements the integrative revision of Lopes-Lima et al. [14] by evaluating the extent to which a geographically expanded dataset based on commonly used mitochondrial markers can recover the broad lineage structure identified using more comprehensive genomic data. The obtained data were further used to investigate the spatial distribution of the identified mitochondrial lineages in relation to the predicted locations of *U. crassus* glacial refugia and the most probable postglacial migration routes of the thick-shelled river mussel. We also compared the recovered mitochondrial lineage pattern with the systematic framework proposed by Lopes-Lima et al. [14], allowing the genetic variation observed in our geographically expanded dataset to be interpreted within the broader evolutionary and biogeographic context of the *U. crassus* complex. In particular, we evaluated whether our sampling provides evidence for previously unreported occurrences or range extensions of mitochondrial lineages, including the lineage corresponding to *U. bruguierianus*. Finally, we discuss the implications of the observed geographic structure for the conservation of evolutionary diversity within the *U. crassus* complex.

## 2. Materials and Methods

### 2.1. Sampling and DNA Isolation

In total, 287 samples of non-gonadal tissue obtained from individuals of *U. crassus* from forty-four rivers in eleven river basins (eight major European river watersheds: Venta, Neman, Vistula, Oder, Vltava, Danube, Dniester, Dnieper and three costal river basins Czorna/Chorna, Słupia and Rega) belonging to the catchments of three seas (Baltic, North, and Black Seas, respectively) were analyzed in the years 2009–2014 (Table S1). 

Sampling sites were selected based on previous data on known locations of *U. crassus* in Central and Southern Europe. Due to the protected status of *U. crassus*, special permits for sample collection in Poland were obtained from the Chief Conservator of Nature (permit no. DOP-OZGIS-4200/1-29/244/10/ed) and the General Director for Environmental Protection (permit no. DOP-OZGIS.6401.10.14.2012.ls). In Ukraine, however, *U. crassus* was not a protected species; therefore, no collection permits were required. For other countries, tissue samples were utilized from *U. crassus* individuals originally collected for different research purposes. In all cases, individuals were generally collected alive and identified based on shell morphology.

Total DNA from 287 individuals was extracted from non-gonadal tissue with a standard phenol–chloroform method after [57], with some modifications. In contrast to the original protocol, the step of the selective precipitation of DNA was modified. Ethyl alcohol (96%) and ammonium acetate (3.0 M) were added in volumes of two and one-tenth volumes of DNA solution, respectively.

To avoid ambiguity, we use the term “sampling locality” to refer to a specific river location from which specimens were collected, whereas “individual” refers to a single *U. crassus* specimen. Sampling localities were distributed across eight river drainages. A total of 44 sampling localities were included in the study, from which 287 individual specimens were collected and subjected to DNA extraction. The numbers of individuals successfully sequenced for each mitochondrial marker may differ among localities. Geographic information for each sampling locality, including river, drainage, country, and coordinates, is provided in Table S1. The haplotypes identified, numbers of corresponding sequences, and GenBank accession numbers are provided in Table S1, whereas sample sizes and summary statistics of genetic polymorphism for each sampling locality are provided in Table S2.

### 2.2. DNA Amplification and Sequencing

For further phylogeographic analyses, two mtDNA markers inherited in the maternal lineage (F-type mtDNA) were chosen: cytochrome oxidase subunit I gene fragment (*cox1*; 553 bp) and mtDNA fragment containing a part of a NADH dehydrogenase subunit 3 gene, five tRNA genes (*tRNA-His, tRNA-Ala*, two *tRNAs-Ser* and *tRNA-Glu*) and a part of dehydrogenase subunit 2 gene (*nadh3*-*tRNA*-*nadh2*; 1292 bp). Amplification of target mtDNA fragments was performed using TProfessional (Biometra, Göttingen-Weststadt, Germany) and MaxyGene^TM^ II (Axygen, Union City, CA, USA) thermal cyclers.

Preliminary amplification of the *cox1* gene fragment was performed using the universal primers HCO2198 and LCO1490 [58]. However, the mitochondrial heteroplasmy, which was observed for the first time in non-gonadal tissue of *U. crassus* by Mioduchowska et al. (2016) [49], was frequently the reason for weak sequencing results. Therefore, the sex-specific primers for amplification of *cox1* only from mtDNA of maternal origin were designed [49]. In the case of *nadh3-tRNA-nadh2*, the primer pair was designed based on the complete F-type mtDNA sequences of the congeneric species *Unio pictorum* [50]. Both molecular markers were amplified for the same individuals.

Amplification of *cox1* gene fragment was performed in a 20 µL volume containing 1x Green GoTaq Flexi Buffer, 2.5 mM of MgCl_2_, 0.2 mM of each dNTP’ (Sigma-Aldrich, part of Merck Life Science, Darmstadt, Germany), 0.4 µM of Fcox1F (5′-AACTATTATCCTGTCACTCAAAAGACGTT-3′) and 0.4 µM of Fcox1R (5′-GAAAGTTTGCGGTGATTGTG-3′) primers, 0.5 U of GoTaq Hot Start Polymerase (Promega, Madison, WI, USA) and ca. 100 ng of DNA. PCR was performed with an initial denaturation step at 95 °C for 2 min followed by 30 cycles at 95 °C for 45 s, 50 °C for 45 s and 72 °C for 1 min 30 s, with a final extension at 72 °C for 5 min.

PCR reactions for amplification of the *nadh3-tRNA-nadh2* fragment were performed in 20 µL reaction volumes containing 1x HF Buffer, 1.5 mM of MgCl_2_, 0.2 mM of each dNTP’ (Sigma-Aldrich, part of Merck Life Science, Darmstadt, Germany), 0.4 µM of ND3F (5′-CACTCATGAACTACCCCAATA-3′) and 0.4 µM of ND2R (5′-GTTGACCCTAATGATTGGG-3′), 0.5 U Polymerase Phusion (Thermo Scientific, Waltham, MA, USA) and ca. 100 ng of DNA. The thermal conditions were as follows: 30 s at 98 °C; 35 cycles of 10 s at 98 °C, 20 s at 61,9 °C, 30 s at 72 °C and a final extension at 72 °C for 7 min. The amplified fragments were visualized by electrophoresis on a 1% agarose gel and purified by exonuclease I (20 U/μL, Thermo Scientific, USA) and alkaline phosphatase FastAP (1 U/μL, Thermo Scientific, Waltham, MA, USA) following the manufacturer’s instructions. Sequences were obtained from both strands using the BigDye sequencing protocol implemented in ABI 3730xl Genetic Analyzer (Macrogen Inc., Seoul, Korea).

### 2.3. Molecular Data Analyses

Sequences were edited and corrected using the BioEdit v. 7.2.5 software [59]. Additional correction of chromatograms and extension of up to 20% of sequences were performed for the *nadh3-tRNA-nadh2* fragments in the PeakTrace application (http://www.nucleics.com/peaktrace/ accessed on 6 October 2026). The identity of the amplified regions was verified using the BLAST 2.17.0 application (Basic Local Alignment Search Tool) and searched against the non-redundant database of the National Center for Biotechnology Information (NCBI) [60]. Consensus sequences were aligned with Clustal Omega [61,62] using default settings for both *cox1* and *nadh3-tRNA*-*nadh2* markers, as well as for concatenated fragments. Additionally, the *cox1* alignment was translated into the corresponding amino acid sequence to check for the presence of stop codons. Haplotypes were determined using the DnaSP v.6.12.03 program [63]. All unique sequences identified for individual markers were deposited in GenBank (accession numbers: KT598034-KT598185; Table S1). For further analyses, sequences of *cox1* and *nadh3*-*tRNA*-*nadh2* markers were concatenated and analyzed as a single locus.

The specimens analyzed in this study were collected between 2009 and 2014, and the corresponding sequences were deposited in GenBank in 2014. Although the molecular data were generated and deposited previously, the present study provides new analyses and comparisons, including with the data published by Lopes-Lima et al. [14].

Genetic polymorphism indices for the concatenated data set, such as the number of unique haplotypes (H), number of polymorphic sites (S), mean number of nucleotide differences (K), haplotype diversity (H_d_), and nucleotide diversity (π), were calculated in DnaSP. Mean genetic distances under the Kimura 2-parameter model (*K2P*) [64] were calculated in MEGA X 10.0.3 [65]. Standard error estimates were obtained by a bootstrap procedure (1000 replicates).

To examine the population structure of *U. crassus*, without making *a priori* assumptions about the partitioning of its genetic diversity, we used the clustering approach implemented in BAPS 6.0 [66,67]. BAPS was run with the number of groups (K) set to 1–10, and each run was replicated 5 times. The spatial distribution of identified clusters was compared with the geographical distribution of the European Union (EU) ecoregions for rivers and lakes (European Environment Agency, https://www.eea.europa.eu/data-and-maps/figures/ecoregions-for-rivers-and-lakes (accessed on 12 May 2025); [68]). The mismatch distribution analyses were determined for BAPS clusters in DnaSP. This analysis uses the minimization of the sum of squared deviations (SSD) between the observed mismatch distribution and its expectation, under the model of sudden expansion, distinguishing the distribution as a result of expansion or from a stable population. To study the hypothesis of a recent demographic expansion, additional analyses were performed using the total number of mutations: Tajima’s *D* [69] and Fu’s *Fs* neutrality tests [70], and Harpending’s raggedness index (*rg*) [71]. The latter quantifies the observed mismatch distribution, showing low values when populations underwent an expansion event, and high when the populations are stable or underwent a bottleneck event. Pairwise genetic differentiation among identified clusters was estimated through the calculation of *F_ST_* values. Tests listed above were calculated in Arlequin 3.5.2.2 [72], and their significance was evaluated using 10,000 simulated samples.

Further analyses focused on determining the relationships among tested samples, as well as dating of *U. crassus* lineage-splitting events. Five data partitions were made (*cox1*, *nadh3*, tRNA, non-coding region and *nadh2*). For each data partition, the best-fitting nucleotide substitution model using the Akaike Information Criterion was estimated using jModelTest 2.1.7 [73]. The *cox1* substitution rate was fixed (0.00265 substitutions per site per million years) following the rate estimated for Unionida by Froufe et al. [23]. This rate was originally estimated for two *Unio* species separated by the Strait of Gibraltar and was used as the reference rate for the *cox1* partition. The substitution rates for the remaining partitions were estimated in BEAST v. 1.8.1 [74] relative to the fixed *cox1* rate. Clock-model selection was initially guided by the posterior distribution of the coefficient of variation (CV) obtained under uncorrelated lognormal relaxed-clock (UCLN) models in BEAST v. 1.8.1. Partitions for which the 95% highest posterior density (HPD) interval of CV was clearly separated from zero were considered to show substantial among-lineage rate heterogeneity, whereas partitions with HPD intervals extending to values close to zero were considered compatible with a strict molecular clock. Accordingly, *cox1* and *nadh2* were assigned relaxed clocks, *nadh3* and the non-coding region strict clocks, while both strict- and relaxed-clock alternatives were retained for the tRNA partition. Four alternative clock configurations were compared: all partitions under strict clocks, all partitions under UCLN relaxed clocks, *cox1* and *nadh2* under relaxed clocks with the remaining partitions under strict clocks, and *cox1*, *nadh2*, and tRNA under relaxed clocks with *nadh3* and the non-coding region under strict clocks. Marginal likelihoods were estimated using stepping-stone sampling in BEAST v. 1.8.1 [75,76], and model support was evaluated using Bayes factors expressed as 2ln(BF), following [77]. Values of 2 < 2ln(BF) < 6, 6 < 2ln(BF) < 10, and 2ln(BF) > 10 were interpreted as positive, strong, and very strong evidence, respectively, against the model with the lower marginal likelihood. The clock configuration supported by the marginal-likelihood and Bayes-factor comparisons was used in the final divergence-time analysis.

The tree prior was set to a constant-size coalescent [78] for all data partitions. Two runs were performed in BEAST, with 20 million generations each and a sampling frequency of 1000. Tracer v. 1.6 [79] was used to analyze log files to assess convergence. The combined effective sample sizes (ESS) for all the chosen parameters were larger than 200. All the obtained trees were combined with LogCombiner v. 1.8.1 [79], with a burn-in of 20%. Finally, the maximum credibility tree was produced using TreeAnnotator v. 1.8.1 [79]. In turn, the reconstruction of genealogical connections and genetic relationships among haplotypes was performed using the PopART 1.7 software with the median-joining algorithm (MJ) applied [80].

Additionally, we analyzed all *cox1* sequences determined in the present study with data described by Lopes-Lima et al. [14] to fit the identified pattern of genetic variation in collected specimens into a broader picture of the recently revised *U. crassus* species complex. An analysis of the extended *cox1* matrix was conducted using MrBayes v. 3.2.5 [81]. The evolutionary model was determined for each codon position using IQ-TREE v. 2.2.2.6 [82] under the Bayesian Information Criterion. The posterior probabilities of clades were estimated by sampling trees from the PP distribution using Markov chain Monte Carlo (MCMC) simulations. Six chains were run for 20,000,000 generations. Trees were sampled every 1000 generations. Convergence of log-likelihood and parameter values from two runs was checked using Tracer 1.7.1 [79]. The first 2500 trees were discarded as the burn-in sample. The remaining trees were used to assess topology, PPs, and gene tree distributions. The final phylogenetic tree was edited in FigTree v. 1.4.4 (http://tree.bio.ed.ac.uk/software/figree/ (accessed on 16 September 2026)).

## 3. Results

### 3.1. Geographic Patterns of Genetic Diversity

A concatenated alignment of 287 sequences (1845 bp; 553 bp for the *cox1* marker and 1292 bp for the *nadh3-tRNA*-*nadh2* marker, respectively) was used for further analyses. The alignment was unambiguous, and the inferred amino acid sequences contained no stop codons, which indicates a mitochondrial origin of those sequences rather than nuclear insertions.

Standard genetic indices calculated for the whole data set are summarized in Table 1. We identified 132 haplotypes, of which 101 (76.5%) haplotypes were represented by a single sequence in the total sample (Table S1). An overall mean genetic distance calculated using the *K2P* model was estimated at 0.0197 ± 0.0022. The same indices were also determined for each collected sample of *U. crassus*. Those values are given in Table S2.

**Figure 1:**
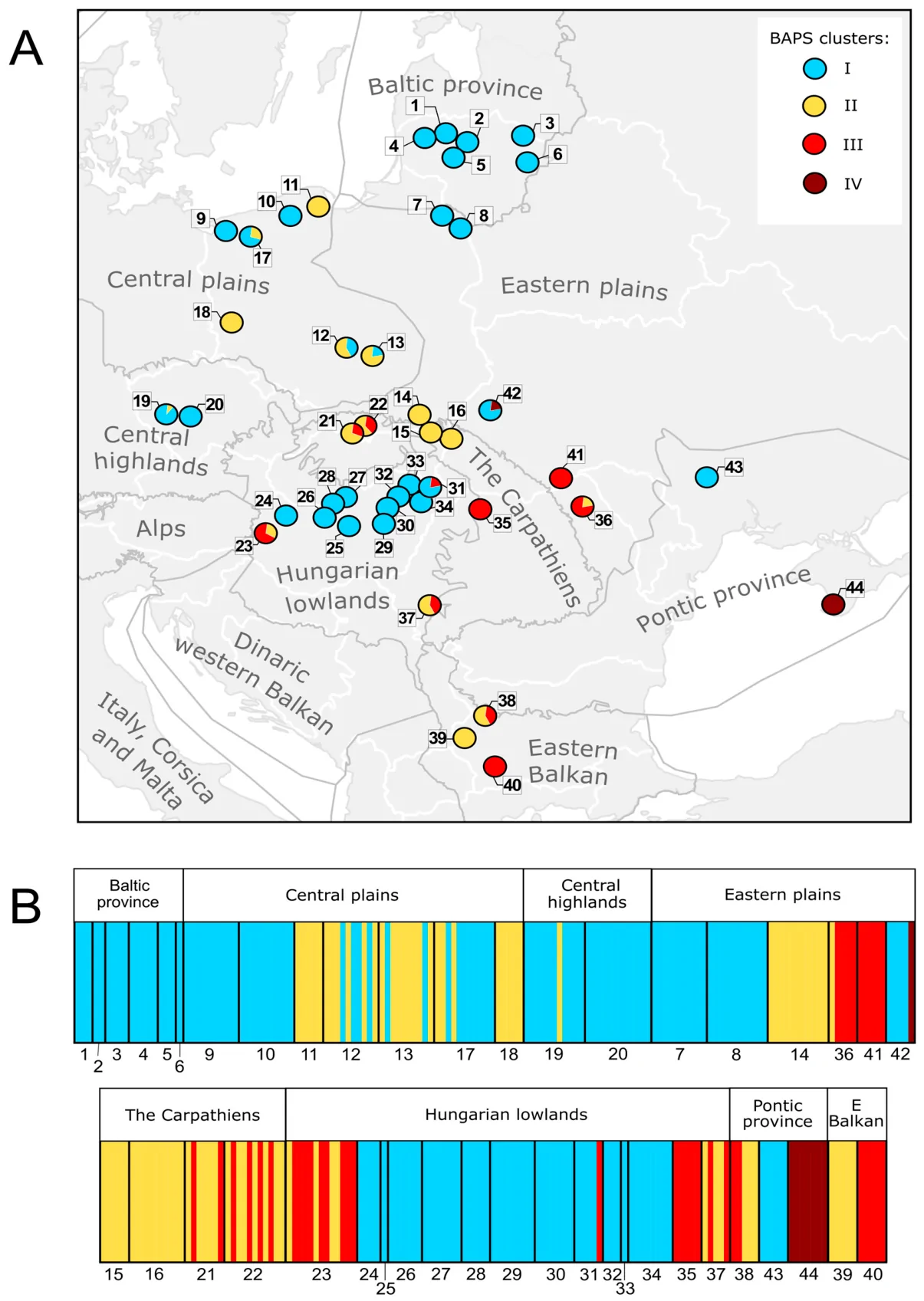
Pattern of the genetic diversity of *Unio crassus* based on the tested mitochondrial markers (combined *cox1* gene fragment and *nadh3*-*tRNA*-*nadh2* region). (**A**) Distribution of Bayesian analysis of population structure-produced clusters within EU ecoregions; (**B**) structure of the identified clusters within EU ecoregions. Pie charts represent the relative proportions (%) of tested individuals assigned to each cluster within individual sampling sites. The total numbers of individuals assigned to the respective clusters were cluster I, n = 158; cluster II, n = 82; cluster III, n = 38; and cluster IV, n = 8. Numbers below plots refer to the selected sampling sites marked on the presented map. Abbreviations: E—eastern.

Mitochondrial data were informative for further clustering using the BAPS coalescent approach, and the analysis of concatenated sequences revealed four separate clusters (I–IV) within *U. crassus* inhabiting rivers in Central and Eastern Europe (Figure 1). Standard genetic indices were also calculated for those clusters (Table 1). Clusters I and III were characterized by the highest H_d_ values (0.947 and 0.940, respectively). In turn, for cluster II, the lowest H_d_ (0.5018) and the lowest mean *K2P* genetic distance (0.0008 ± 0.0001) values were observed. In the case of cluster IV, the mean *K2P* value reached the highest value (0.0112 ± 0.0019) among the analyzed clusters and, at the same time, a low H_d_ (0.679) and a high nucleotide diversity (π) (0.011).

The *K2P* genetic distance calculated for pairs of identified clusters was in the range 0.7–4.1% (Table 2). The lowest value was observed between clusters II and III, while the highest genetic distance was observed between clusters I and IV. Values of *K2P* genetic distance calculated for other pairs of clusters were *c.* 3% (Table 2).

Further analysis revealed the spatial distribution pattern of the four identified clusters. Cluster I occurred across almost the entire studied area (apart from the southernmost sites; Figure 1) and extended into the Hungarian Lowlands (the Pannonian Basin). Cluster II occurred mainly in Central Europe, whereas in Southern Europe, two clusters dominated: cluster III occurred on the Balkan Peninsula and in areas near the Carpathian massif, while cluster IV occurred mainly on the Crimean Peninsula (Figure 1). Pairs of different clusters occurred in sympatry at 12 of the 44 sampling localities across Central and Southeastern Europe, representing approximately 27% of the sampling sites (Figure 1). The sympatric occurrence of haplotypes assigned to more than two clusters was not observed.

### 3.2. Network Analysis

**Figure 2:**
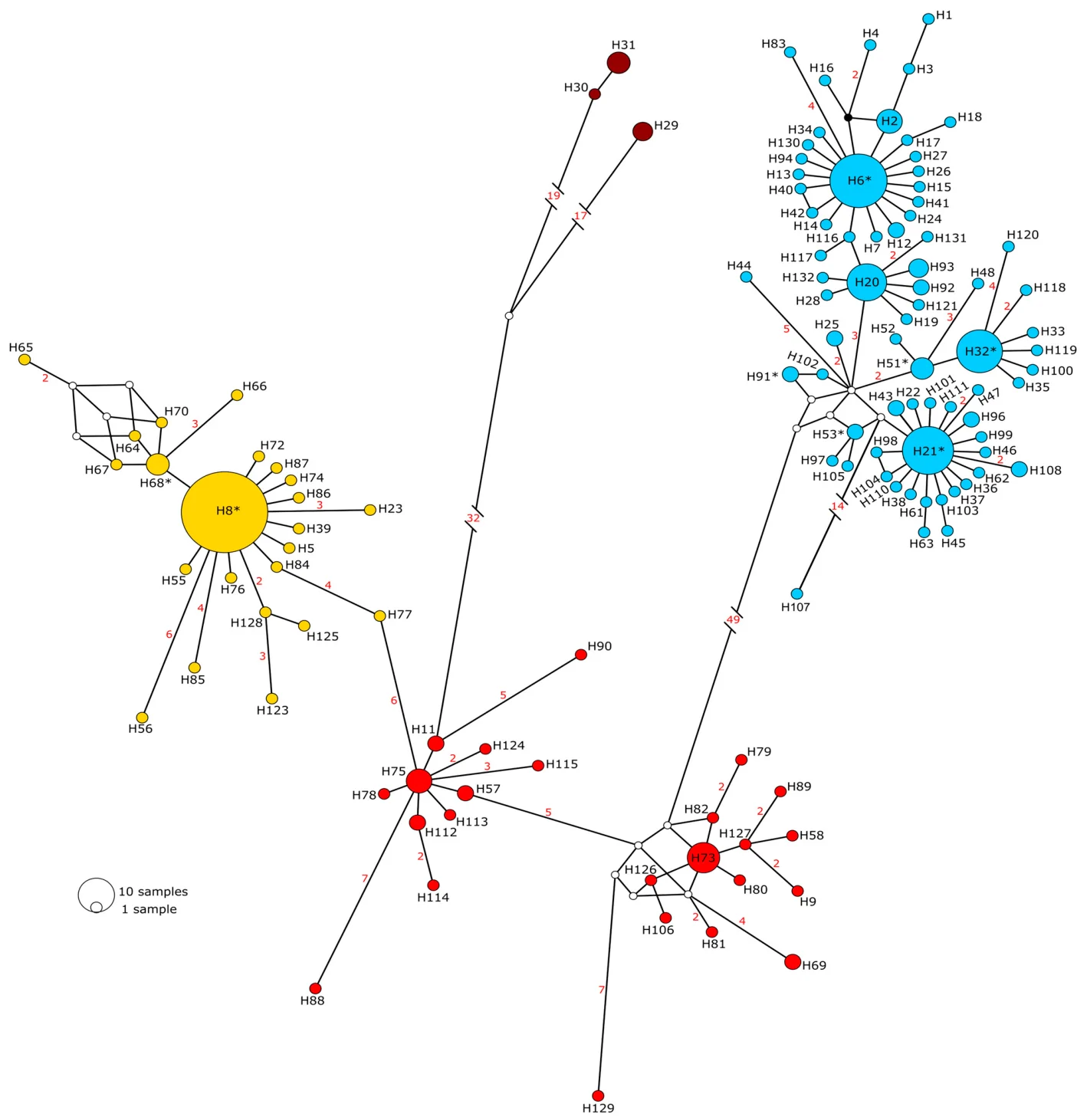
Median-joining network of concatenated haplotypes including *cox1* gene fragment and *nadh3-tRNA-nadh2* region. White circles indicate median vectors (ancestral, unsampled or extinct haplotypes). Numbers at the branches denote the number of mutational steps between haplotypes (values above 1 are shown). Circle sizes are proportional to the haplotype frequency. Asterisks indicate haplotypes merged with the most similar sequences during analysis in PopART. Color scheme reflects the BAPS clustering.

Separate haplogroups corresponding to clusters identified in BAPS were also observed in the median-joining network (Figure 2). As a result, some haplotypes were merged with the most similar sequences (marked with asterisks). Groups I-III were composed of multiple closely related haplotypes forming characteristic star-like topologies, i.e., a multifurcation with many short branches connected at the internal (ancestral) node. In the large and haplotype-rich group I, central haplotypes H6* (H6+H95) and H32* (H32+H50) were found mainly in Northern Poland and Lithuania, and haplotype H20 was noticed in the eastern range of the area covered by that haplogroup. In turn, haplotype H21* (H21+H49) was observed predominantly in the Hungarian Lowlands. In group II, central haplotype H8* (H8+H10) was noticed throughout the group’s area, apart from Eastern Balkan, where H68* (H68+H71) was identified. In the latter region, we also observed the presence of H73—one of the two central haplotypes identified for haplogroup III. H73 was also found in samples collected further north (Rába in Hungary and Grónik in Southern Poland). Within that region, we observed the other central haplotype identified in haplogroup III—H75, occurring also in Siret (Romania) and in Prut (Ukraine). Although the star-like topology was found for haplogroup III, haplotypes incorporated into that group were more differentiated, and frequencies of central ones were lower than those identified in haplogroups I and II. Haplotypes clustered in haplogroup IV did not form a star-like topology, but two of them (H29 and H31) were more numerous.

### 3.3. Time-Calibrated Reconstruction of Phylogeny

Tracer diagnostics revealed clear differences in among-lineage rate variation among partitions (Table 3). Substantial rate heterogeneity was observed for *cox1* and *nadh2*, supporting relaxed-clock models for these partitions, whereas *nadh3* and the non-coding region were compatible with strict clocks. The tRNA partition showed an intermediate pattern and was therefore retained under both strict- and relaxed-clock alternatives for subsequent model comparison.

Stepping-stone marginal-likelihood comparisons further differentiated the alternative clock configurations (Table 4). The all-relaxed model and the mixed model with relaxed clocks for *cox1*, *nad2*, and tRNAs received essentially equivalent support. In contrast, assigning a strict clock to tRNAs reduced model support, while the all-strict model was strongly disfavoured. Given the negligible difference between the two best-supported models, the less complex mixed-clock configuration was selected for the final divergence-time analysis. The direct comparison of the two mixed-clock models also favored a relaxed clock for the tRNA partition (2lnBF = 5.63), providing positive evidence for this configuration.

**Figure 3:**
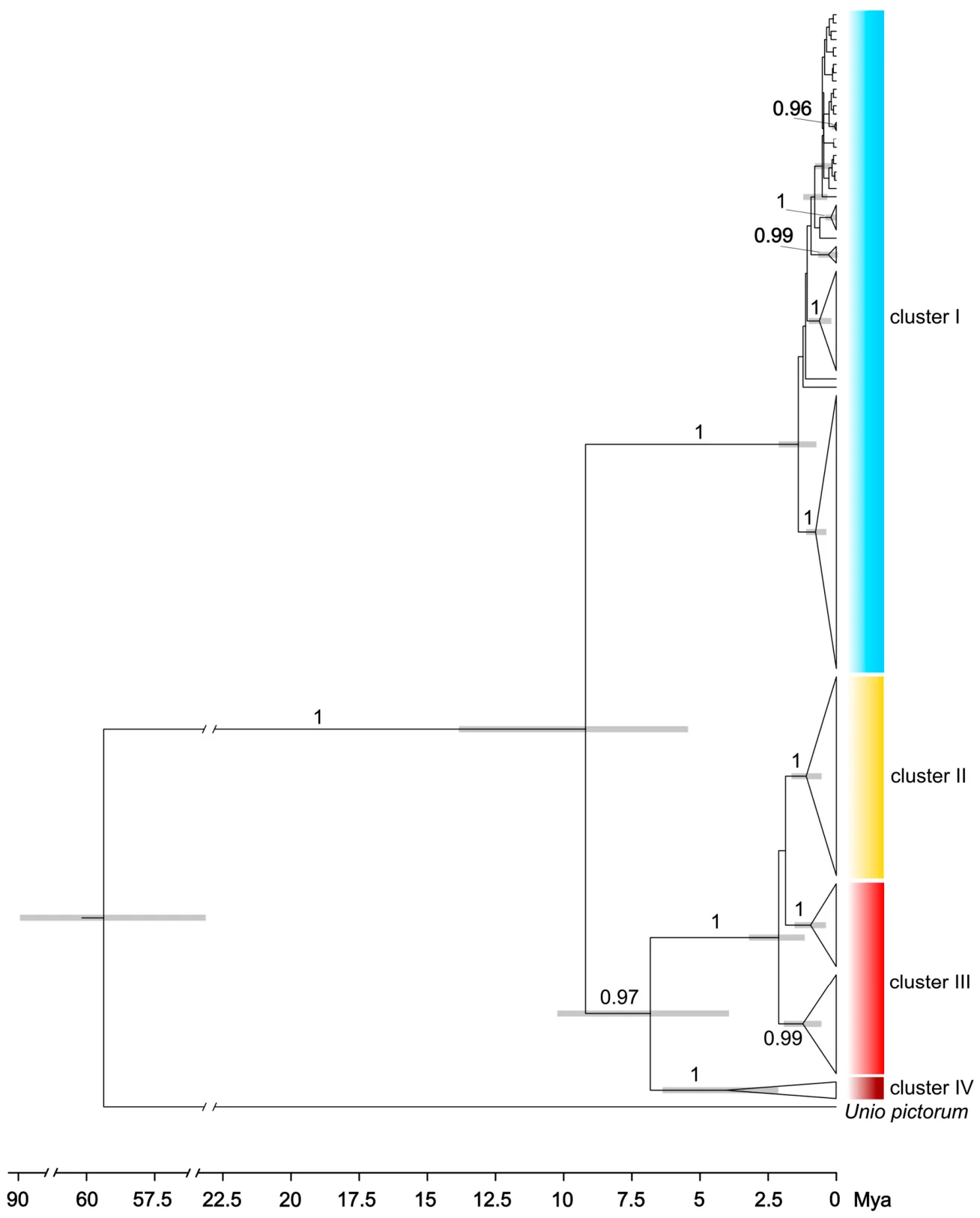
Bayesian chronogram based on the concatenated *cox1* + *nadh3-tRNA-nadh2* fragments, showing relationships among the identified clusters. Node values indicate Bayesian posterior probabilities > 0.95. Node bars represent the 95% highest posterior density (HPD) intervals for node ages. Triangle size reflects the diversity within clades.

Our time-calibrated phylogeny reconstruction on concatenated *cox1* + *nadh3-tRNA-nadh2* fragments revealed the presence of five, well-supported haplogroups congruent with those identified by network analysis (Figure 3). Obtained results suggest that *U. crassus* started to diverge in the late Miocene (*c.* 9.2 Ma; 95% HPD: 5.44–13.85) when the split of the lineage leading to clusters I and II–IV took place. The second lineage-splitting event was dated at the turn of the Miocene and Pliocene (*c.* 6.82; 95% HPD: 3.93–10.23). At that point in time, lineages leading to the merged clusters II and III, and cluster IV, originated. The most basal cluster IV was composed of haplotypes from the Crimean Peninsula, in the Ponto-Caspian Province. Cluster II formed a strongly supported monophyletic group, whereas cluster III was divided into two strongly supported clades and was not monophyletic. However, clusters II and III together constituted a strongly supported monophyletic group (Figure 3 and Figure S1). Cluster II is distributed more northwards in the central part of the selected sampling region (mostly in Poland and northern parts of the Carpathian Mountains), while cluster III seems to be distributed more southwards (near the Eastern Carpathian Mountains and in the Balkans). Cluster II seems to have originated in the Pleistocene (*c.* 2.12 Ma; 95% HPD: 1.16–3.2).

### 3.4. Gene Flow Estimation and Analyses of Demographic History

A comparison of pairwise *F_ST_* values indicated that all clusters determined by Bayesian analysis of population structure were significantly different from each other (Table 5). Selective neutrality was estimated by Tajima’s *D* and Fu’s *F_S_* tests. Those statistics were negative for clusters I, II, and III, but for the latter one, the value of Fu’s *F_S_* test was not as negative as for clusters I and II, and it was not statistically significant (Table 6). Negative values of neutrality tests may be consistent with recent demographic expansion. In turn, both tests calculated for cluster IV were positive and insignificant.

The low values of raggedness index calculated for clusters I–III, in the range of 0.00929–0.07588 (Table 6), were also compatible with an expansion model. However, the corresponding values were not statistically significant, and therefore do not provide independent strong support for a demographic expansion.

**Figure 4:**
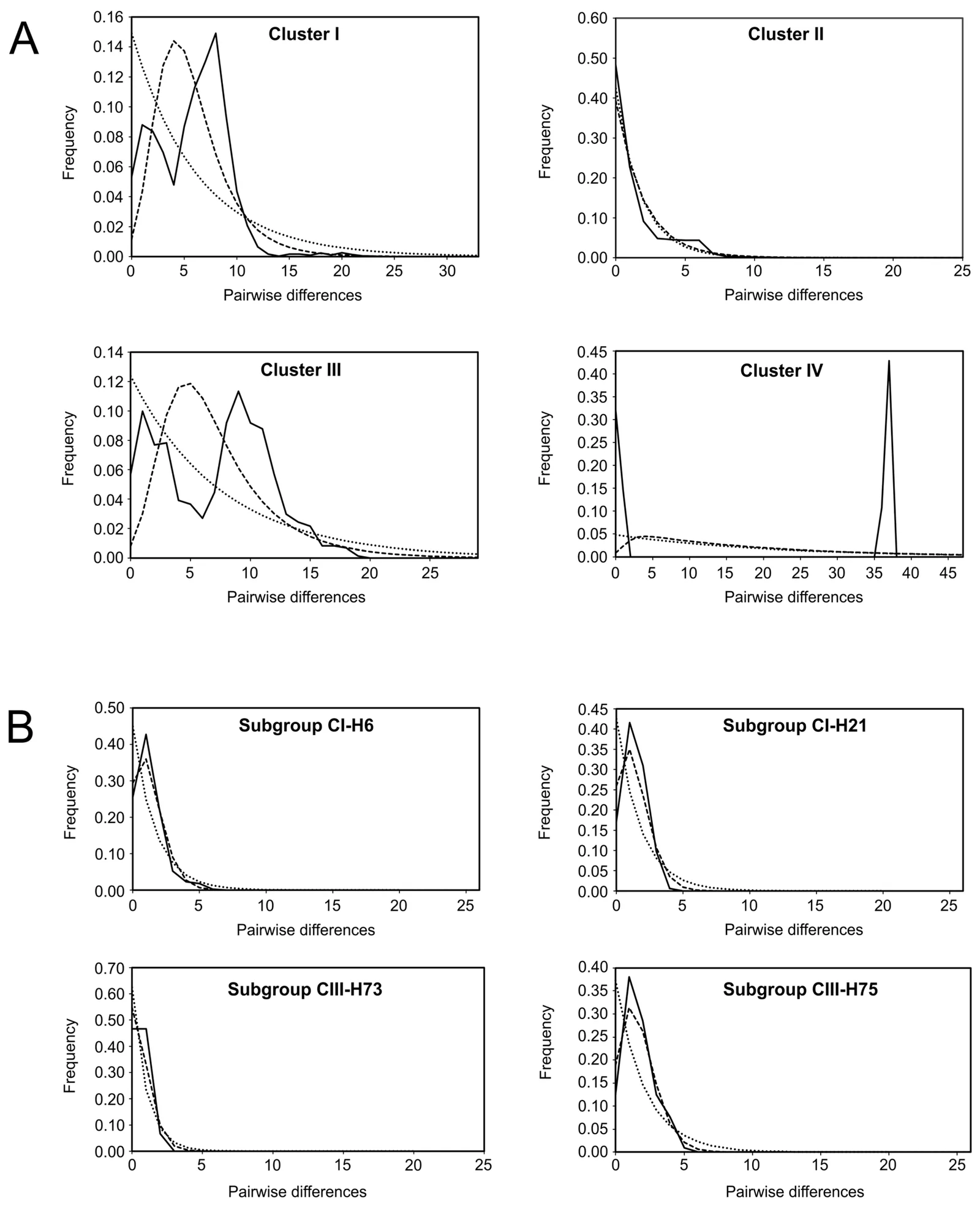
Mismatch distribution (pairwise differences). Graphs represent the mismatch distributions determined for (**A**) clusters I–IV identified using Bayesian analysis of population structure, and (**B**) subgroups identified within clusters I and III. Dotted and dashed lines indicate expected distributions under constant size and expansion models, respectively. Solid line indicates observed distributions.

Population size changes were also tested for the four clusters determined by Bayesian analysis of population structure. A skewed unimodal distribution was observed only for cluster II, which is consistent with a recent sudden expansion of this group (Figure 4A). In contrast, bimodal distributions were observed for clusters I, III, and IV. Such a pattern may be associated with relatively stable population size, but may also result from the presence of two genetically differentiated lineages within a cluster. Therefore, the bimodal distributions should not be interpreted as direct evidence of demographic stability. To further investigate this pattern, data for clusters I and III were subdivided according to the topology of the haplotype network (Figure 4B). Additional mismatch distributions were calculated for subgroups CI-H6 and CI-H21 within cluster I (including central haplotypes H6 and H21, respectively, and haplotypes directly linked with them), and subgroups CIII-H73 and CIII-H75 within cluster III (subgroup CIII-H73 included haplotypes H73, H80, H82, and H127; subgroup CIII-H75 included haplotypes H11, H57, H75, H78, H112, H113, H115, and H124). Due to insufficient sample size, cluster IV was excluded from further separation into subgroups.

Further analyses yielded unimodal distributions for all identified subgroups (Figure 4B). The *F_ST_* values between those subgroups within clusters were estimated as 0.88040 (*p* = 0.00000) for the CI-H6–CI-H21 pair, and 0.92768 (*p* = 0.00000) for the CIII-H73–CIII-H75 pair, respectively.

**Figure 5:**
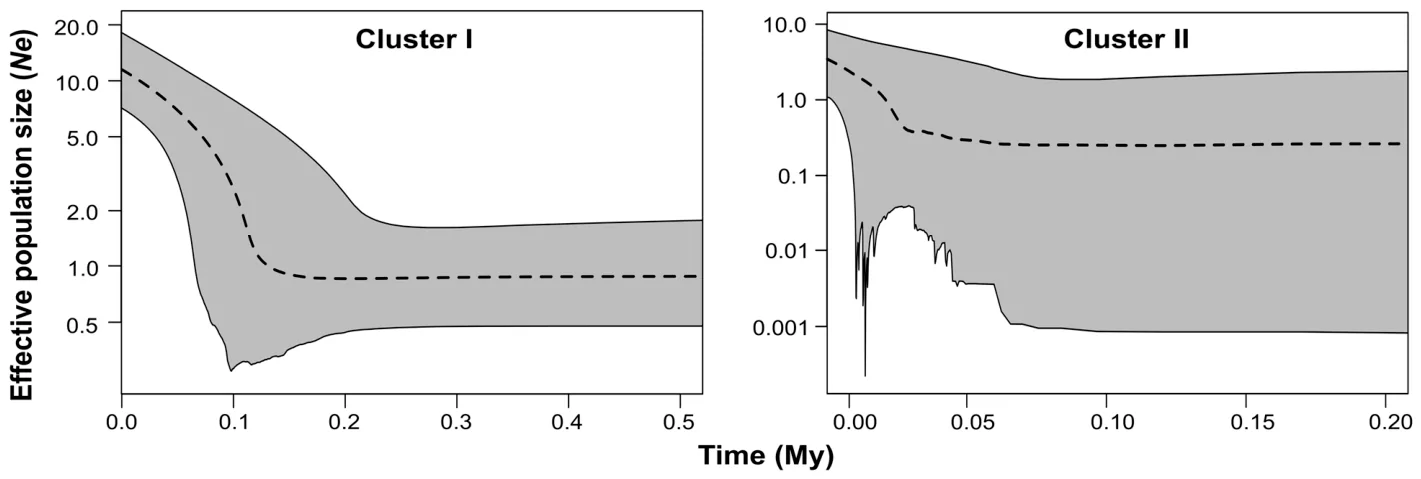
Extended Bayesian skyline plots (eBSP) based on concatenated mitochondrial data and determined for clusters I and II, respectively. Plots determined for two other identified clusters are presented in Figure S1.

Additionally, we determined eBSP plots for each identified cluster (Figure 5 and Figure S2). For clusters I and II, the eBSPs were consistent with an increase in effective population size during the Pleistocene, estimated at approximately 125 ka for cluster I and 25 ka for cluster II, respectively. By contrast, the population size within cluster III appeared to be relatively stable, with no clear signs of sudden expansion (Figure S1). However, the flat eBSP pattern may have resulted from the limited sample size and the consequently reduced power to detect demographic expansion. In cluster IV, the eBSP plot indicated a decline in effective population size (Figure S2). However, this pattern should be interpreted with caution because cluster IV comprised highly divergent haplotypes and had a strongly limited sample size. These factors may have resulted in an erroneous inference of demographic decline.

By contrast, the population size within cluster III seems to be more or less stable, without signs of sudden expansion (Figure S1). However, flat eBSP may be rather the result of insufficient sample size and a lack of power to detect the expansion. In turn, a decline in effective population size found in the eBSP plot determined for cluster IV may be recognized as an artifact (Figure S2). That cluster is composed of highly divergent haplotypes and its sample size is strongly limited. Those two reasons may lead to an erroneous inference of demographic decline within cluster IV.

### 3.5. Comparative Analysis with Data Determined for the Unio Crassus Species Complex

**Figure 6:**
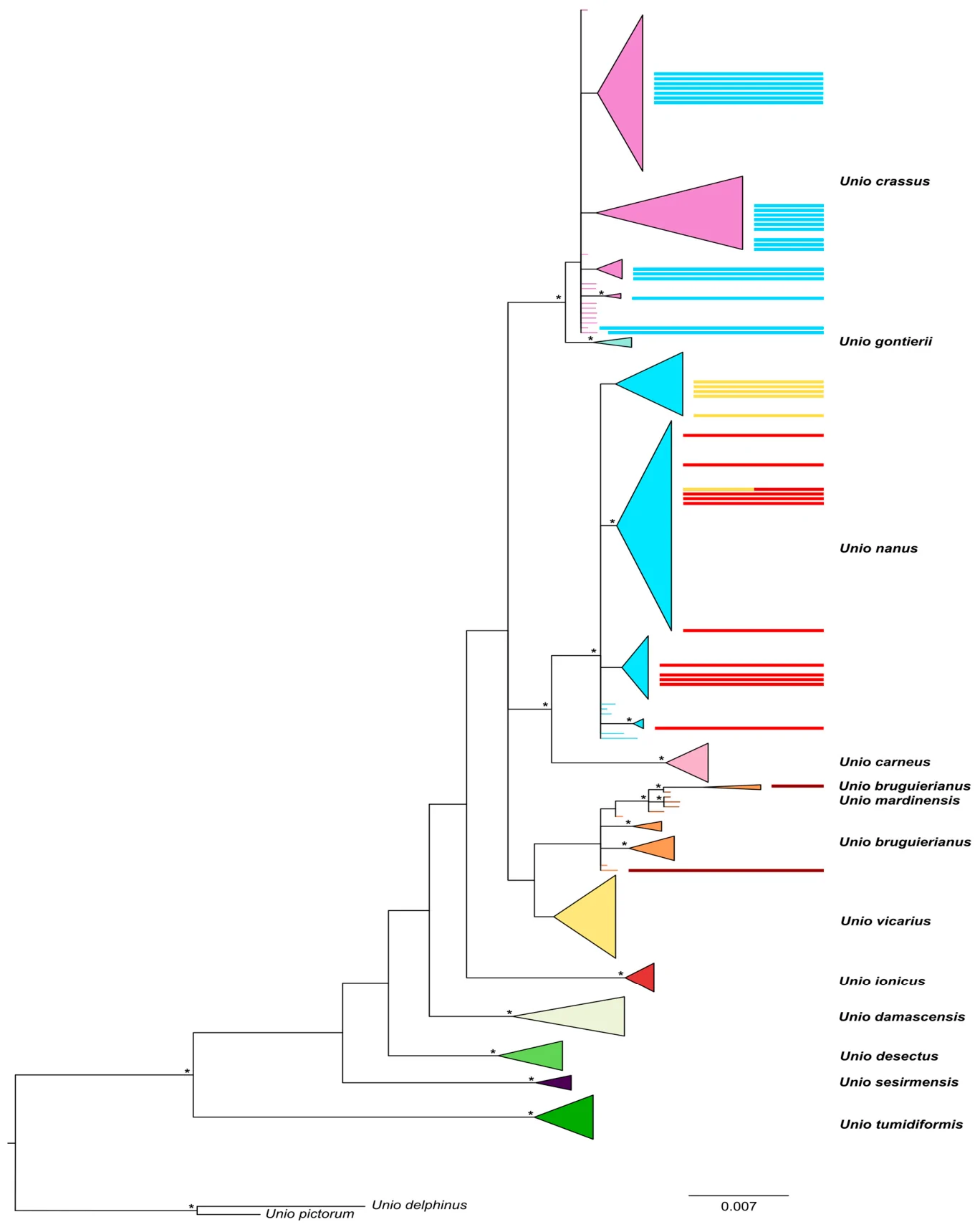
Bayesian phylogenetic tree inferred from the *cox1* gene fragment for haplotypes determined in the present study and additional haplotypes obtained for species belonging to the *Unio crassus* complex. Support values > 0.95 are indicated by an asterisk. Colors of triangles refer to the color scheme given by Lopes-Lima et al. [14]. Horizontal bars indicate haplotypes determined in the present study with reference to the identified clusters (blue—cluster I, yellow—cluster II, red—cluster III, and burgundy—cluster IV).

The *cox1* haplotypes determined in the present study were analyzed together with data obtained from a recent report on the genetic diversity within the *U. crassus* species complex [14]. The topology of the final Bayesian tree as well as posterior probability values were congruent with those determined in the mentioned study (Figure 6).

Selected fragments of the *cox1* gene were less variable than *nadh3-tRNA-nadh2*. However, the pattern of clustering was maintained, and all but one haplotype determined here were associated with a single cluster. Only one *cox1* haplotype (marked as “99”; Table S3, Figure S3) refers to two BAPS clusters (II and III, respectively).

The comparative analysis revealed an association between the genetic clusters identified here and the species delineated within the *Unio crassus* complex (Figure 6). However, the identified clusters were interpreted as mitochondrial lineages corresponding to clades associated with the species entities recognized by Lopes-Lima et al. [14], without making definitive species-level assignments. Interestingly, haplotypes associated with cluster I were grouped within the *U. crassus* s. str. clade. Both clusters II and III, which showed a more subtle separation in the BAPS analysis based on concatenated *cox1* and *nadh3-tRNA-nadh2* sequences, were placed within the *U. nanus* clade in the comparative phylogenetic analysis. In turn, two *cox1* haplotypes associated with cluster IV were grouped with haplotypes belonging to the *U. bruguierianus* clade. However, the position of one of these haplotypes within the *U. bruguierianus* clade was not well supported. Therefore, its phylogenetic position should be interpreted with caution and does not, by itself, provide sufficient evidence for considering it a separate or novel lineage within *U. bruguierianus*.

Furthermore, we compared geographic ranges determined for those three species belonging to the *U. crassus* complex with the pattern of spatial distribution of distinct clusters determined in the present study. The distributions of cluster I and combined clusters II and III fit into the species range of *U. crassus* s. str. and *U. nanus*, respectively. In turn, cluster IV was associated with *U. bruguierianus*, which has not been found in the Crimean Peninsula.

## 4. Discussion

The genus *Unio* is among the most widespread freshwater mussel taxa in the world [21,83]. Moreover, it remains particularly notorious for its intraspecific phenotypic plasticity and regional variation in shell shape [23]. Thus, up to the 20th century, over 1000 described species were included within the genus [84]. Haas [85] revised the *Unio* representatives and considered the genus as a series of 12 “fundamental” *Unio* species with many subspecies or races. More recently, results of molecular studies allowed the taxonomy of the genus to be revised, resulting in about 25 currently recognized extant *Unio* species [86]. Nevertheless, within some morphospecies (e.g., *U. gibbus*, *U. tigridis* or *U. crassus*) distinct phylogenetic lineages have also been recognized [21]. Taking into account the fact that freshwater mussels are among the most threatened fauna, the proper analyses of the genetic variation within *Unio* species and accurate identification of intraspecific lineages are essential for the development of effective conservation strategies(e.g., [44]).

In the present study, we detected a pronounced pattern of mitochondrial genetic differentiation among populations of freshwater mussels identified based on shell morphology as *U. crassus* and sampled across Central and Eastern Europe. This genetic structure is consistent with the substantial diversity previously reported within the *U. crassus* complex [21,42,43,45,46,49,54,87], while the broad overlap in shell morphospace among several members of the complex limits the reliability of morphology-based identification [14]. Lopes-Lima et al. [14] subsequently provided an integrative revision of the complex based on mitochondrial and nuclear data, recognizing 12 geographically structured species. Rather than independently reassessing species boundaries, our study provides a geographically expanded mitochondrial dataset that allows us to examine whether the broad lineage structure identified by the integrative revision can also be recovered using the *nadh3–tRNA–nadh2* and *cox1* markers. The concordance between the mitochondrial lineages recovered in our study and the broad structure reported by Lopes-Lima et al. [14] supports the utility of these markers for detecting major lineage-level differentiation within the complex, while our additional sampling contributes new information on the geographic distribution of these lineages in Central and Eastern Europe. Thus, our results complement rather than replicate the integrative revision of Lopes-Lima et al. [14] by extending the geographic coverage and providing an assessment of mitochondrial lineage structure based on markers that can be more readily applied to regional and population-level datasets.

An additional contribution of the present study concerns the geographic distribution of the mitochondrial lineage corresponding to *U. bruguierianus*. The detection of this lineage further northwards, including also the Crimean Peninsula and at least the Dniester River Basin, extends its previously reported geographic distribution and provides additional information on the spatial structure of the *U. crassus* complex. However, given that our analyses are based exclusively on maternally inherited mitochondrial markers, this correspondence should be interpreted at the mitochondrial lineage level rather than as independent species-level confirmation. Further analyses incorporating nuclear markers would be required to evaluate the extent to which this mitochondrial lineage corresponds to the species boundaries proposed by Lopes-Lima et al. [14].

Analyses based on two selected mitochondrial markers (*cox1* and *nadh3-tRNA*-*nadh2*) allowed us to identify four well-supported groups (i.e., clusters I–IV). We calculated the matrix of *K2P* among these clusters, and the values were in the range 0.7–4.1%. The lowest value was calculated for clusters II and III, for which the separation visible both on the dendrogram and the haplotype network was more subtle. Values of *K2P* distance calculated for other compared clusters were higher (>3.0%). Similar values were obtained by Araujo et al. (2018) [21] among several lineages identified within *U. crassus* during analyses based on the *cox1* mitochondrial marker. In that study, the authors analyzed the genetic diversity of samples collected mainly from European rivers (but also from the Asian part of Türkiye). As a result, samples from rivers located in France, Germany, Slovakia, Greece, and Türkiye have been separated into at least six distinct groups representing different lineages. Further analyses of species delimitation have been performed, but the results obtained were inconsistent and revealed two to six main lineages/species with no clear geographic structuring. However, as mentioned above, a more recent integrative approach performed on a wide spatial scale allowed confirmation of the complex status of *U. crassus* with 12 geographically structured species [14]. The genetic distances among those species (mostly >3%, with an average *cox1* divergence between all species estimated as 4.9% ± 2.1%) were similar to values calculated between clusters identified here. Intraspecific distances above 3% were also noticed among other *Unio* representatives [22]. Thus, we performed further analyses involving sequences obtained in the present study and data determined in the mentioned research on the *U. crassus* species complex. Although these analyses were based only on the *cox1* mitochondrial marker, we obtained a strict association between haplotypes belonging to clusters I–IV and species representing the *U. crassus* complex. Interestingly, only haplotypes associated with cluster I grouped within the *U. crassus* s. str. clade. Clusters II and III, whose separation was more subtle, were placed within the *U. nanus* clade. In turn, two *cox1* haplotypes associated with the clade IV grouped with haplotypes belonging to the *U. bruguierianus* clade.

We also compared interspecific genetic distances calculated for those three species with values determined for identified clusters. Corresponding values were similar and lower than 1%. Interestingly, clusters II and III identified by BAPS analysis differed by only 0.7%, and this value was slightly higher than the intraspecific *p*-distance calculated for *U. nanus* (0.6%). Although values of the genetic distances close to 1% were also noticed between the mitochondrial lineage associated with *U. bruguierianus* and *U. madrinensis* or *U. crassus* and *U. gontierii* species, and were explained as resulting from mitochondrial introgression or incomplete lineage sorting, we believe that both clades II and III refer to *U. nanus* and the genetic distance between them reflects intraspecific diversity of this mitochondrial lineage. Moreover, the genetic distance close to 1% was also observed within cluster IV, referred to as *U. bruguierianus*. Nevertheless, the value was highly similar to that calculated by Lopes-Lima et al. (2024) [14] for *U. bruguierianus,* indicating its significant intraspecific divergence, visible also in the topology of the phylogenetic tree.

Taking into account the separation determined for samples analyzed in the present study, which is in line with the pattern of delimited species within the *U. crassus* complex, we compared the divergence times estimated for both distinct genetic clusters and identified the mitochondrial lineage. Our estimations were based on two mitochondrial markers, but time-calibrated phylogeny determined for the *U. crassus* species complex were based on full sequences of mitogenomes. Although we assumed that mitogenome-based estimations of divergence time could be more precise, both results are congruent. In turn, the indicated congruence shows that two mitochondrial markers selected in the present study (*cox1* and newly determined *nadh3*-*tRNA-nadh2*) are sufficient to make estimates comparable to those derived from advanced mitogenome sequencing techniques.

In the present study, we have also attempted to determine the ranges of distribution of the identified clusters. Cluster I was widely distributed and occurred almost throughout the studied area (with an exception to its southernmost parts). This spatial distribution fits into the distribution range of corresponding *U. crassus* s. str. The mentioned species occurs in most Atlantic and Baltic Sea river basins, as well as in river basins in Eastern Europe extending to the Caspian and Black Sea basins [14]. In turn, the distribution of combined clusters II and III fits into the mitochondrial lineage range of corresponding *U. nanus*. Cluster II was present mainly in Central Europe, whereas cluster III occurred predominantly in Southern Europe on the Balkan Peninsula and areas near the Carpathian massif. *U. nanus* is widely distributed but restricted to Europe, with a core distribution in the Saône sub-catchment of the Rhône, the Rhine, and the Danube basins. The range of the mitochondrial lineage extends from these basins to the English Channel, North Sea, and Baltic Sea basins, from the Seine in the west to the Vistula in the east.

The last cluster identified in the present study (cluster IV) referred to *U. bruguierianus*. Its distribution range is more limited, and individuals belonging to this mitochondrial lineage inhabit rivers in the Western Anatolian region of Türkiye, north of the Büyük Menderes and west of the Sakarya River basins. *U. bruguierianus* distribution extends to the Aegean and Black Sea river basins of European Türkiye, Greece, and Bulgaria. However, in the present study, specimens corresponding to this mitochondrial lineage were collected on the Crimean Peninsula (from the Czorna River) and the western part of Ukraine (from the Czerkawa River), where *U. bruguierianus* has not been reported. According to Haas [86], the Crimean Peninsula is inhabited by *U. gontierii*, the other species belonging to the *U. crassus* complex. However, Lopes-Lima et al. [14] pointed out that the mentioned report was prepared without molecular background and thus, it should be taken with caution. In turn, Western Ukraine is covered by overlapping ranges of *U. crassus* s. str. and *U. nanus*. Therefore, we believe that our molecular study provides additional and valuable data that allow us to extend the distribution range of *U. bruguierianus* further north, including also the Crimean Peninsula and at least the Dniester River Basin. At the same time, we cannot unambiguously exclude the possibility that *U. gontierii* occurs on the Crimean Peninsula, as proposed by Lopes-Lima et al. [14]. The number of specimens collected from this area and analyzed in the present study was limited. Thus, we cannot exclude that the ranges of *U. bruguierianus* and *U. gontierii* overlap in that area.

Shevchuk et al. [46] reported two taxa within the *Unio crassus* complex from Ukraine, *U. crassus* s. str. and *U. stevenianus* (taxon name according to Zhadin [88]). They were described using morphological features and two molecular mtDNA markers, *cox1* (572 bp) and 16S rRNA (471 bp). *U. stevenianus* was found in the Chorna River on the Crimean Peninsula and in the Borzhava River in the Danube basin (Carpathian foothills). The reanalysis showed that it is *U. bruguierianus*, confirming our results. In turn, *U. crassus* s. str. was found in the Sluch River in the north, and in the Siversky Donets River in the east of Ukraine, but also in the Karasu River on the Crimean Peninsula, showing a wide range of the mitochondrial lineage.

The clear separation of distinct clusters identified here, as well as—on a wider scale—the recognition of 12 species within the *U. crassus* complex, raises questions about their evolutionary history, particularly in the context of potential glacial refugia and postglacial migration routes. Well-known glacial refugia were located predominantly in Southern Europe (mainly on the Mediterranean peninsulas, but also in the Pannonian Basin, the Ponto-Caspian region, and the Carpathians) [89,90,91,92,93,94,95,96]. Isolation in such areas caused ongoing population divergence through genetic drift and local selection, contributing to the formation of distinct phylogenetic lineages [97]. Following the retreat of the ice sheet, populations subsequently expanded their ranges into formerly glaciated areas, recolonizing Europe northwards [91,92,98].

The potential locations of the glacial refugia inhabited by particular mitochondrial lineage species can be inferred from the spatial distribution of their genetic diversity. These areas are typically expected to harbor higher intraspecific genetic diversity than the surrounding recolonized regions. The explanation of that pattern assumes that populations surviving in the glacial refugia accumulated genetic variation, while it decreased in leading-edge populations during the postglacial recolonization processes [97]. Feind et al. [37] analyzed the genetic population structure of *U. crassus* samples collected in Germany and Sweden. Based on a set of nine microsatellite markers, the authors identified three major conservation units for the analyzed *U. crassus* populations and revealed the presence of two putative glacial refugia of this species (i.e., Elbe River basin and Danube-Rhine system).

In the present study, the genetic distances among the identified clusters and mitochondrial lineages within the *U. crassus* complex, together with their phylogenetic separation, are consistent with a history of prolonged lineage differentiation. The independence of the four clusters was also supported by high and significant values of the F*_ST_* index, although the genetic distance between clusters II and III was relatively low (*c.* 0.7%). These results suggest that the four clusters may have experienced partly distinct phylogeographic histories. However, the demographic scenarios proposed below should be regarded as hypotheses rather than definitive reconstructions, because they are based primarily on mitochondrial variation and are therefore potentially affected by the particular inheritance pattern and demographic history of the mitochondrial genome.

Our mitochondrial data allow us to propose as a working hypothesis that the identified groups originated from three main refugial areas and that the deep divergence among currently observed lineages within the *U. crassus* complex occurred in the Ponto-Caspian region and areas surrounding the Black Sea. Based on our data, the main split between the observed groups likely took place in the late Miocene, approximately 9.20 Ma (95% HPD: 5.44–13.85 Ma), when the lineage leading to cluster I (*U. crassus* s. str.) diverged from the lineage comprising clusters II–IV, and approximately 6.82 Ma (95% HPD: 3.93–10.23 Ma), when the lineage leading to cluster IV (*U. bruguierianus*) diverged from that comprising clusters II and III (*U. nanus*). These estimates are broadly consistent with those reported by Lopes-Lima et al. [14] based on complete mitogenome sequences, who dated the corresponding divergence events to approximately 6 Ma and 5 Ma, respectively. Although our point estimates are older, both values reported by Lopes-Lima et al. [14] fall within the 95% HPD intervals obtained in the present study.

During that time, the Ponto-Caspian region was subject to significant climatic fluctuations and sea level changes, including changes in connectivity between the Caspian and Black Sea basins [99] (Ref. [100] and references therein). Approximately 4.15 million years ago, according to a comprehensive reconstruction of palaeoecological conditions, there was a transition from hypersaline marine to fluviolacustrine freshwater environments associated with the progressive filling of the basin [100]. Such environmental changes may have contributed to population fragmentation and lineage differentiation. In this context, the present distribution of clusters I (assigned to *U. crassus* s str.), II + III (assigned to *U. nanus*), and IV (assigned to *U. bruguierianus*) is compatible with the hypothesis that their ancestral populations occupied different areas of Southern and Southeastern Europe. Interestingly, refugia of freshwater fish species (also well-known and important hosts of *U. crassus* immature stages [101,102] like the chub *Squalius cephalus* [103] and the bullhead *Cottus gobio* [104]) have been identified in those two regions. Nevertheless, the precise locations of ancestral populations for determined clusters and the subsequent routes of range expansion cannot be determined from the present mitochondrial dataset alone.

The divergence between clusters II and III, interpreted here as intraspecific differentiation within *U. nanus*, was estimated in the Pleistocene at *c.* 2.12 Ma. Several features of the mitochondrial molecular data, including the star-like structure of the haplotype network, neutrality statistics, mismatch distribution, and eBSP results, are compatible with past demographic expansion within cluster II. The estimated timing of this expansion was approximately 25 ka, before the Last Glacial Maximum when southern and central parts of Europe were not covered by the ice sheet [105]. However, these results should be interpreted as evidence consistent with, rather than demonstrating, a demographic expansion. This applies in particular to cluster III, represented by a comparatively limited number of samples, which reduces the statistical power of demographic analyses and limits the reliability of inferences about its demographic history.

Cluster I (fitting into *U. crassus* s. str.) was widely distributed and occurred across most of the studied area. Although that group was especially abundant in the proglacial part of Europe, it was also present in the Pannonian Basin. As mentioned above, we suggest the pre-Pleistocene origin of the cluster I and its expansion in Pleistocene. The estimated timing of approximately 125 ka is consistent with the Eemian interglacial period [106,107]. The haplotype network showed several star-like structures, and the demographic analyses were compatible with a possible past expansion.

Identified star-like structures belonging to the subclades seen in the tree topology differed in their spatial distribution. In general, haplotypes included in star-like topology with H21* central haplotype occurred almost exclusively in the Pannonian Basin. In turn, three other such topologies (with H6*, H20, and H32* central haplotypes) included haplotypes distributed predominantly in northern parts of the studied area (in Northern Poland and Lithuania). The concentration of genetic diversity in the Pannonian Basin, together with the spatial distribution of several common haplotypes, is also compatible with the hypothesis that this region may have played a role in the historical persistence and subsequent spread of the cluster I. However, the available data do not allow us to identify the Pannonian Basin unequivocally as a glacial refugium.

The spatial distribution of the identified clusters is consistent with a scenario of postglacial range expansion and recolonization of areas previously affected by ice sheet advance. Although the northwards migration routes of freshwater fauna were primarily dependent on the rivers′ reshaping and rearrangement of water connections, the spatial pattern of recolonization may also be impacted by the migration of *U. crassus* immature stages through meltwater connections along the ice sheet margins. We hypothesize that these expansion routes were plausible, given that one of the most suitable hosts of *U. crassus* glochidia, the common minnow (*Phoxinus phoxinus*), is a well-known cold-adapted fish species. These fishes are able to survive adverse cold climatic conditions and are very adaptable to various stream and lake habitats as well as plastic with regard to dietary preferences [108]. Nevertheless, the contribution of host-mediated dispersal to the specific phylogeographic patterns observed here remains hypothetical and requires further investigation using independent nuclear markers and broader population sampling.

We assume that similar patterns of the spatial distribution of the genetic variation could also be found for other freshwater species widely distributed in Europe. At the same time, our findings suggest that complexes of cryptic species may be more numerous within the family Unionidae and deep divergences within other species could represent a broader evolutionary trend. Three well-supported clades have also been reported in the freshwater mussel *Anodonta anatina* [109]. The distribution of its clade 2 broadly corresponded to that of clusters I and II in *U. crassus*. Subsequent studies confirmed four distinct *A. anatina* lineages, with the most widely distributed lineage further divided into European and Eurasian sub-lineages, while the remaining lineages were geographically restricted [110,111]. These patterns were interpreted as reflecting the persistence of genetic diversity in multiple glacial refugia.

Distinct but closely related mitochondrial lineages have also been identified in *Margaritifera margaritifera*, with Northern and Southern European distributions [19,112]. Genetic similarity between North American and Northeastern European populations has further been linked to historical postglacial dispersal [113]. A similar two-lineage pattern was reported in the European bitterling, *Rhodeus amarus*, whose Central and Western European lineages broadly corresponded to the distribution of *U. crassus* s. str. and *U. nanus* [114]. As *U. crassus* is a host for *R. amarus* [115], this correspondence may partly reflect their ecological association. Similar postglacial patterns have also been reported in other freshwater species. In *Asellus aquaticus*, mitochondrial and nuclear data revealed genetic clusters associated with two major postglacial regions differing in their climatic history [116]. The spatial distribution of these clusters broadly resembled the pattern observed in *U. crassus*, with clusters II–IV showing similarities to lineages associated with periglacial regions, whereas cluster I more closely corresponded to the widespread proglacial group. The results described in the present study confirm that the thick-shelled river mussel is not a genetically homogeneous mitochondrial lineage. Taking into account its decline within its range, identifying distinct phylogenetic lineages corresponding to cryptic mitochondrial lineages and assessing their genetic diversity are important steps for success in conservation management efforts. Without the use of molecular data, it would have been difficult to identify the diversity within the thick-shelled river mussel, and the results will be critical to the success of conservation management plans.

Overall, the phylogeographic structure of *U. crassus* likely reflects the combined effects of historical climatic processes, population history, environmental conditions, and host-associated microorganisms. Previous studies have shown that Pleistocene climatic fluctuations and postglacial colonization shaped the genetic diversity and spatial structure of *U. crassus* s. st. and *U. nanus* populations in Europe [55,56]. In this context, the association between phylogeography and the gut microbiome of *U. crassus* is noteworthy. Mioduchowska et al. [117] found that geographically isolated populations differed in gut microbiome composition, which was more strongly associated with phylogeographic structure than with local habitat conditions. In addition, *Cardinium* and *Wolbachia* endosymbionts have been detected in *U. crassus* [118,119]. As maternally inherited endosymbionts may affect host reproduction and mitochondrial diversity, their presence should be considered when interpreting mitochondrial phylogeographic patterns.

Taken together, our findings confirm that *U. crassus* sensu lato is genetically structured and that the mitochondrial diversity observed across its range is consistent with the presence of several distinct evolutionary lineages. The spatial distribution of these lineages, together with the results of demographic analyses, provides indications of complex historical processes involving lineage differentiation, population persistence, and more recent range expansion. Nevertheless, the demographic scenarios proposed here remain tentative, because they are inferred primarily from mitochondrial variation and the strength of demographic signals varies among clusters. In particular, the small sample size of cluster IV and the limited statistical power for cluster III preclude robust conclusions about their demographic histories.

Molecular data are therefore important for recognizing evolutionary diversity within the *U. crassus* species complex, but a more comprehensive reconstruction of its demographic and phylogeographic history will require, e.g., additional nuclear markers. Integrating mitochondrial and nuclear data with ecological and environmental information would provide a stronger basis for testing hypotheses concerning glacial refugia, postglacial expansion, and population connectivity, and would ultimately improve the assessment of evolutionary diversity relevant to conservation management. An important limitation of the present study is that the inference of taxonomic identity is based exclusively on maternally inherited mitochondrial markers. Although the mitochondrial lineages identified here correspond closely to the clades associated with species entities recognized by Lopes-Lima et al. [14], mitochondrial data alone cannot conclusively establish species boundaries. In particular, incomplete lineage sorting, mitochondrial introgression, and sex-associated mitochondrial inheritance may lead to discordance between mitochondrial lineages and species boundaries. Therefore, the species-level interpretation of the lineages identified in the present study should be considered provisional and would benefit from independent nuclear evidence.

## 5. Conclusions

The endangered thick-shelled river mussel appears to reflect a complex evolutionary history evident in its genetic diversity and evolutionary patterns. Our results are in line with a recent study on the *U. crassus* species complex. According to both studies, the main divergence most likely took place during the late Miocene and early Pliocene. Further processes led to the differentiation of four separate clusters/lineages with distinct spatial distributions in Central and Eastern Europe, corresponding to *U. crassus* s. str., *U. nanus*, and *U. bruguierianus*. As a working hypothesis, we propose that these groups are related to distinct glacial refugia located in the peripheral part of the Ponto-Caspian region surrounding the Crimean Peninsula, in the Eastern Balkans, and in the Pannonian Basin. Moreover, the study provides new data on the distribution range of *U. bruguierianus*, which has not been previously reported from the Crimean Peninsula and the Dniester River Basin. These records should be regarded as a tentative range extension, pending morphological re-examination of the available specimens and confirmation from additional material from the Black Sea basin.

## Figures and Tables

**Table 1:** Standard genetic indices calculated for the whole data set and for determined separate clusters.

Data Type	Sample Size (n)	Nucleotide Diversity (π)	Haplotype Diversity (Hd)	Number of Unique Haplotypes (H)	Number of Polymorphic Sites (S)	Mean Number of Nucleotide Differences (K)	Mean *K2P* Genetic Distance ± S.E.
Whole data set	287	0.01902	0.943	132	233	34.063	0.0197 ± 0.0022
Cluster I	158	0.00317	0.947	74	97	5.710	0.0032 ± 0.0007
Cluster II	82	0.00075	0.518	23	40	1.361	0.0008 ± 0.0001
Cluster III	38	0.00392	0.940	23	50	7.068	0.0039 ± 0.0009
Cluster IV	8	0.01100	0.679	3	37	19.857	0.0112 ± 0.0019

**Table 2:** The matrix with values of *K2P* genetic distances calculated for pairs of determined genetic clusters. Values of the genetic distances are given below the diagonal, and S.E. values are given above the diagonal.

	Cluster I	Cluster II	Cluster III	Cluster IV
Cluster I		0.0045	0.0042	0.0044
Cluster II	0.0351		0.0016	0.0039
Cluster III	0.0330	0.0072		0.0037
Cluster IV	0.0405	0.0321	0.0307	

**Table 3:** Clock-model selection based on Tracer diagnostics.

Partition	Mean CV	95% HPD of CV	Clock Model
*cox1*	1.74	0.98–2.57	Relaxed (UCLN)
*nadh2*	1.89	0.69–3.32	Relaxed (UCLN)
*nadh3*	0.26	~0–0.76	Strict
tRNAs	0.52	~0–1.23	Strict/Relaxed (UCLN)
non-coding region	0.25	~0–0.72	Strict

**Table 4:** Comparison of alternative molecular-clock configurations using stepping-stone marginal likelihoods and Bayes factors.

Clock-Model Configuration	Log Marginal Likelihood	2ln(BF) vs. Best Model	Interpretation
All partitions relaxed (UCLN)	−6374.749	-	Highest marginal likelihood
*cox1*, *nadh2*, *tRNA*s relaxed; *nadh3* and non-coding region strict *	−6374.823	0.15	No meaningful difference from the best model
*cox1* and *nadh2* relaxed; *nadh3*, *tRNA*s and non-coding region strict	−6377.641	5.78	Positive evidence against this model
All partitions strict	−6397.073	44.65	Very strong evidence against this model

Note. Bayes-factor interpretation follows Kass and Raftery: 2 < 2ln(BF) < 6, positive evidence; 6 < 2ln(BF) < 10, strong evidence; and 2ln(BF) > 10, very strong evidence against the model with the lower marginal likelihood. The direct comparison of the two mixed-clock models yielded 2ln(BF) = 5.63 in favor of the model with a relaxed clock for tRNAs. *—model selected for analysis.

**Table 5:** The matrix of pairwise *F_ST_* (below diagonal) and *p*-values (above diagonal) among four groups identified using Bayesian analysis of population structure.

	Cluster I	Cluster II	Cluster III	Cluster IV
Cluster I	-	0.00000	0.00000	0.00000
Cluster II	0.90490	-	0.00000	0.00000
Cluster III	0.85624	0.69470	-	0.00000
Cluster IV	0.86843	0.94320	0.75072	-

**Table 6:** Values of Tajima’s *D* and Fu’s *F_S_* neutrality tests, and raggedness index with corresponding *p*-values calculated for identified clusters.

Cluster	Tajima’s D		Fu’s F_S_		Raggedness Index	
	*D*	*p*	F_S_	*p*	Rg	*p*
I	−2.10932	0.00160	−24.30530	0.00030	0.00929	0.70000
II	−2.65432	0.00000	−25.76698	0.00000	0.07588	1.00000
III	−1.47778	0.04480	−2.65036	0.19630	0.01321	0.70000
IV	2.08997	0.99820	9.69405	0.99940	0.35077	0.15000

## Data Availability

The data that support the findings of this study are openly available in the GenBank database under accession numbers KT598034–KT598185.

## Supplementary Materials

The following supporting information can be downloaded at: https://www.mdpi.com/article/10.3390/genes17101282/s1, Table S1: Overview of sampling locations, unique *Unio crassus* haplotypes, their cluster classification, and GenBank accession numbers. Abbreviations: B.S.—Baltic Sea, N.S.—North Sea, Bl.S.—Black Sea; country codes: LT—Lithuania, PL—Poland, CZ—Czech Republic, H—Hungary, R—Romania, BG—Bulgaria, UA—Ukraine. Table colors indicate haplotype cluster assignments: blue (cluster I), yellow (cluster II), red (cluster III), and brown (cluster IV). The number of sequences corresponds to the number of individuals. The same site numbers, haplotype codes, and cluster colors are applied to the other figures. Table S2: DNA polymorphism data for collected individuals of *Unio crassus*. Table S3: List of *cox1* haplotypes identified for tested specimens and individuals included in the study on the *Unio crassus* complex. The reference to the species, as well as to the accession numbers, is given. Color boxes refer to the distinct clusters identified in the present study (blue—cluster I, yellow—cluster II, red—cluster III, burgundy—cluster IV) based on sequences of the combined *cox1* gene fragment and *nadh3-tRNA-nadh2* region [120]. Figure S1: Bayesian chronogram constructed for concatenated *cox1* + *nadh3-tRNA-nadh2* fragments, showing the relationships among the identified haplotypes. Node values indicate Bayesian posterior probabilities > 0.95. Node bars represent the 95% highest posterior density (HPD) intervals for node ages. Figure S2: Extended Bayesian skyline plots (eBSP) based on concatenated mitochondrial data and determined for clusters III and IV, respectively. Figure S3: Detailed Bayesian phylogenetic tree inferred from the *cox1* gene fragment for haplotypes determined in the present study and additional haplotypes obtained for species belonging to the *Unio crassus* complex. All included haplotypes are listed in Table S3. Support values > 0.95 are indicated by an asterisk. Colors of vertical bars refer to the color scheme given by Lopes-Lima et al. [14]. Horizontal bars indicate haplotypes determined in the present study with reference to the identified clusters (blue—cluster I, yellow—cluster II, red—cluster III, and burgundy—cluster IV).
