# Supplementary material for: Unveiling the Identity of Unio crassus (Bivalvia: Unionidae) in Central and Eastern Europe
Source: Genes (Basel). 2026 Oct 14;17(10):1282. doi: 10.3390/genes17101282 (PMC13704050; doi:10.3390/genes17101282)
Supplement: Supplementary file 1 [file genes-17-01282-s001.zip › Table_S1.pdf]

Table S1. Overview of sampling locations, unique *Unio crassus* haplotypes, their cluster classification, and GenBank accession numbers. Abbreviations: B.S. – Baltic Sea, N.S. – North Sea, Bl.S. – Black Sea; country codes: LT – Lithuania, PL – Poland, CZ – Czech Republic, H – Hungary, R – Romania, BG – Bulgaria, UA – Ukraine. Table colors indicate haplotype cluster assignments: blue (cluster I), yellow (cluster II), red (cluster III), and brown (cluster IV). The number of sequences corresponds to the number of individuals. The same site numbers, haplotype codes, and cluster colors are applied to the other figures.

No. (sampling site)	River (code)	River basin (catchment of the sea)	Country	GPS coordinates		Cluster	Haplotype code (number of sequences)	*cox1*	*nd3-tRNA-nd2*
				° N	° E				
1.	Virvyčia (VIR)	Venta (B.S.)	LT	55.955	22.504	I	H20 (1)	KT598039	KT598080
						I	H131 (1)	KT598039	KT598184
						I	H132 (1)	KT598039	KT598185
2.	Dubissa (DUB)	Neman (B.S.)	LT	55.703	23.082	I	H21 (1)	KT598040	KT598094
						I	H118 (1)	KT598071	KT598104
3.	Zalvė (ZAL)	Neman (B.S.)	LT	55.821	25.898	I	H32 (2)	KT598044	KT598104
						I	H121 (1)	KT598039	KT598087
						I	H122(1)	KT598039	KT598175
4.	Babrungas (BAB)	Neman (B.S.)	LT	55.936	21.892	I	H32 (3)	KT598044	KT598104
						I	H93 (1)	KT598039	KT598154
						I	H120 (1)	KT598072	KT598104
5.	Šešuvis (SES)	Neman (B.S.)	LT	55.442	22.868	I	H32 (1)	KT598044	KT598104
						I	H93 (1)	KT598039	KT598154
						I	H119 (1)	KT598044	KT598174
6.	Luknelė (LUK)	Neman (B.S.)	LT	55.212	25.886	I	H130 (1)	KT598034	KT598183
7.	Szeszupa (SZE)	Neman (B.S.)	PL	54.271	22.912	I	H6 (1)	KT598034	KT598080
						I	H32 (5)	KT598044	KT598104
						I	H60 (1)	KT598044	KT598126
						I	H93 (1)	KT598039	KT598154
						I	H94 (1)	KT598034	KT598155
						I	H95 (1)	KT598034	KT598156
8.	Czarna Hańcza (CZH)	Neman (B.S.)	PL	53.971	23.304	I	H6 (1)	KT598034	KT598080
						I	H20 (1)	KT598039	KT598080
						I	H32 (3)	KT598044	KT598104
						I	H33 (1)	KT598044	KT598105
						I	H34 (1)	KT598034	KT598106
						I	H35 (1)	KT598045	KT598107
						I	H36 (1)	KT598046	KT598094
						I	H37 (1)	KT598040	KT598108
						I	H38 (1)	KT598040	KT598109
9.	Brzeźniacka Węgorza (BWE)	coastal Rega (B.S.)	PL	53.552	15.674	I	H6 (4)	KT598034	KT598080
						I	H20 (2)	KT598039	KT598080
						I	H24 (1)	KT598034	KT598097
						I	H25 (1)	KT598039	KT598098
						I	H26 (1)	KT598034	KT598099
						I	H27 (1)	KT598034	KT598100
10.	Słupia (SLP)	coastal (B.S.)	PL	54.250	17.580	I	H6 (6)	KT598034	KT598080
						I	H25 (1)	KT598039	KT598098
						I	H91 (1)	KT598039	KT598152
						I	H92 (2)	KT598039	KT598153
11.	Radunia (RAD)	Vistula (B.S.)	PL	54.306	18.307	II	H10 (4)	KT598035	KT598084
						II	H85 (1)	KT598035	KT598146
12.	Pilica	Vistula	PL	50.907	19.794	I	H6 (3)	KT598034	KT598080

	(PIL)	(B.S.)				I	H83 (1)	KT598060	KT598080
						II	H10 (5)	KT598035	KT598084
						II	H84 (1)	KT598061	KT598084
13.	Warkocz (WAR)	Vistula (B.S.)	PL	50.841	20.758	I	H116 (1)	KT598069	KT598080
						I	H117 (1)	KT598070	KT598080
						II	H10 (8)	KT598035	KT598084
14.	Jasiołka (JAS)	Vistula (B.S.)	PL	49.693	21.670	II	H10 (9)	KT598035	KT598084
						II	H55 (1)	KT598052	KT598084
						II	H56 (1)	KT598053	KT598123
15.	Osławica (OSL)	Vistula (B.S.)	PL	49.354	22.090	II	H10 (4)	KT598035	KT598043
						II	H72 (1)	KT598035	KT598138
16.	San (SAN)	Vistula (B.S.)	PL	49.218	22.718	II	H10 (8)	KT598035	KT598084
						II	H86 (1)	KT598035	KT598147
						II	H87 (1)	KT598035	KT598148
17.	Drawa (DRW)	Oder (B.S.)	PL	53.496	15.810	I	H6 (4)	KT598034	KT598080
						I	H20 (1)	KT598039	KT598080
						I	H40 (1)	KT598034	KT598111
						I	H41 (1)	KT598047	KT598080
						I	H42 (1)	KT598034	KT598112
						II	H10 (2)	KT598035	KT598084
						II	H39 (1)	KT598035	KT598110
18.	Brzeźniczanka (BRZ)	Oder (B.S.)	PL	51.779	15.271	II	H10 (4)	KT598035	KT598084
						II	H23 (1)	KT598035	KT598096
19.	Chumava (CHU)	Vltava (N.S.)	CZ	49.869	14.003	I	H1 (1)	KT598034	KT598075
						I	H2 (5)	KT598034	KT598076
						I	H3 (1)	KT598034	KT598077
						I	H4 (1)	KT598034	KT598078
						I	H6 (1)	KT598034	KT598080
						I	H7 (1)	KT598034	KT598081
						II	H5 (1)	KT598035	KT598079
20.	Sázava (SAZ)	Vltava (N.S.)	CZ	49.838	14.675	I	H6 (4)	KT598034	KT598080
						I	H12 (2)	KT598034	KT598086
						I	H13 (1)	KT598034	KT598087
						I	H14 (1)	KT598034	KT598088
						I	H15 (1)	KT598034	KT598089
						I	H16 (1)	KT598038	KT598090
						I	H17 (1)	KT598034	KT598091
						I	H18 (1)	KT598034	KT598092
21.	Jeleśnia (JEL)	Danube (Bl.S.)	PL	49.404	19.682	II	H10 (5)	KT598035	KT598084
						III	H57 (1)	KT598054	KT598085
						III	H58 (1)	KT598055	KT598124
22.	Grónik (GRN)	Danube (Bl.S.)	PL	49.465	19.767	II	H10 (4)	KT598035	KT598043
						II	H74 (1)	KT598035	KT598140
						II	H76 (1)	KT598035	KT598141
						II	H77 (1)	KT598057	KT598142
						III	H57 (1)	KT598054	KT598085
						III	H73 (1)	KT598055	KT598139
						III	H75 (1)	KT598057	KT598085
						III	H78 (1)	KT598058	KT598085
23.	Rába[1] (URA)	Danube (Bl.S.)	H	47.348	17.013	II	H10 (1)	KT598035	KT598084
						II	H123 (1)	KT598073	KT598176
						II	H125 (1)	KT598073	KT598178
						II	H128 (1)	KT598073	KT598181
						III	H11 (1)	KT598037	KT598085
						III	H73 (2)	KT598055	KT598139
						III	H75 (2)	KT598057	KT598085
						III	H124 (1)	KT598057	KT598177
						III	H126 (1)	KT598055	KT598179

						III	H127 (1)	KT598055	KT598180
						III	H129 (1)	KT598074	KT598182
24.	Rába[2] (GYO)	Danube (Bl.S.)	H	47.676	17.619	I	H21 (1)	KT598040	KT598094
						I	H49 (2)	KT598040	KT598117
						I	H50 (1)	KT598044	KT598118
25.	Danube (GOD)	- (Bl.S.)	H	47.695	19.129	I	H48 (1)	KT598044	KT598116
26.	Ipoly[1] (LET)	Danube (Bl.S.)	H	47.889	18.762	I	H21 (1)	KT598040	KT598094
						I	H59 (1)	KT598044	KT598125
						I	H60 (1)	KT598044	KT598126
						I	H61 (1)	KT598040	KT598127
						I	H62 (1)	KT598040	KT598128
						I	H63 (1)	KT598040	KT598129
27.	Ipoly[2] (IPV)	Danube (Bl.S.)	H	48.066	19.107	I	H21 (3)	KT598040	KT598094
						I	H51 (1)	KT598044	KT598119
						I	H52 (1)	KT598044	KT598120
						I	H53 (1)	KT598051	KT598121
						I	H54 (1)	KT598051	KT598122
28.	Ipoly[3] (TES)	Danube (Bl.S.)	H	48.010	18.819	I	H21 (2)	KT598040	KT598094
						I	H101 (1)	KT598040	KT598161
						I	H102 (1)	KT598039	KT598162
						I	H103 (1)	KT598064	KT598094
29.	Tisza[1] (TIP)	Danube (Bl.S.)	H	47.889	21.061	I	H21 (2)	KT598040	KT598094
						I	H107 (1)	KT598065	KT598094
						I	H108 (2)	KT598040	KT598166
						I	H109 (1)	KT598040	KT598167
						I	H110 (1)	KT598066	KT598168
						I	H111 (1)	KT598040	KT598169
30.	Tisza[2] (TAK)	Danube (Bl.S.)	H	48.043	21.227	I	H21 (1)	KT598040	KT598094
						I	H96 (2)	KT598040	KT598157
						I	H97 (1)	KT598063	KT598121
						I	H98 (1)	KT598040	KT598158
						I	H99 (1)	KT598040	KT598159
						I	H100 (1)	KT598044	KT598160
31.	Tisza[3] (TIB)	Danube (Bl.S.)	H	48.165	21.661	I	H21 (1)	KT598040	KT598094
						I	H50 (1)	KT598044	KT598118
						I	H104 (1)	KT598040	KT598163
						I	H105 (1)	KT598051	KT598164
						III	H106 (1)	KT598055	KT598165
32.	Bodrog[1] (BOK)	Danube (Bl.S.)	H	48.170	21.363	I	H21 (3)	KT598040	KT598094
33.	Bodrog[2] (BOL)	Danube (Bl.S.)	H	48.276	21.508	I	H22 (1)	KT598040	KT598095
34.	Bodrog[3] (FLB)	Danube (Bl.S.)	H	48.045	21.689	I	H21 (2)	KT598040	KT598094
						I	H43 (2)	KT598048	KT598094
						I	H44 (1)	KT598049	KT598113
						I	H45 (1)	KT598050	KT598094
						I	H46 (1)	KT598040	KT598114
						I	H47 (1)	KT598040	KT598115
35.	Talna (TAL)	Danube (Bl.S.)	R	47.889	23.230	III	H112 (2)	KT598057	KT598170
						III	H113 (1)	KT598057	KT598171
						III	H114 (1)	KT598067	KT598172
						III	H115 (1)	KT598068	KT598173
36.	Siret (SIR)	Danube (Bl.S.)	R	47.702	26.457	II	H71 (1)	KT598035	KT598137
						III	H75 (1)	KT598057	KT598085
						III	H88 (1)	KT598062	KT598149
						III	H89 (1)	KT598055	KT598150
						III	H90 (1)	KT598037	KT598151

37.	Bega (BEG)	Danube (Bl.S.)	R	45.832	22.006	II	H8 (1)	KT598035	KT598082
						II	H10 (2)	KT598035	KT598043
						III	H9 (1)	KT598036	KT598083
						III	H11 (1)	KT598037	KT598085
38.	Ogosta (OGO)	Danube (Bl.S.)	BG	43.406	23.055	II	H70 (1)	KT598035	KT598136
						II	H71 (2)	KT598035	KT598137
						III	H69 (2)	KT598056	KT598135
39.	Nishava (NIS)	Danube (Bl.S.)	BG	43.002	22.891	II	H64 (1)	KT598035	KT598130
						II	H65 (1)	KT598035	KT598131
						II	H66 (1)	KT598035	KT598132
						II	H67 (1)	KT598035	KT598133
						II	H68 (1)	KT598035	KT598134
40.	Palakariya (PAL)	Danube (Bl.S.)	BG	42.370	23.422	III	H73 (5)	KT598055	KT598139
41.	Prut (PRT)	Danube (Bl.S.)	UA	48.341	25.705	III	H75 (1)	KT598057	KT598085
						III	H79 (1)	KT598059	KT598143
						III	H80 (1)	KT598055	KT598144
						III	H81 (1)	KT598055	KT598145
						III	H82 (1)	KT598059	KT598139
42.	Czerkawka (CZE)	Dniester (Bl.S.)	UA	49.420	23.225	I	H20 (3)	KT598039	KT598080
						I	H28 (1)	KT598041	KT598080
						IV	H29 (1)	KT598042	KT598101
43.	Boh (BOH)	Dnieper (Bl.S.)	UA	48.130	30.552	I	H19 (1)	KT598039	KT598093
						I	H20 (4)	KT598039	KT598080
44.	Czorna (CZO)	coastal (Bl.S.)	UA	44.542	33.664	IV	H29 (2)	KT598042	KT598101
						IV	H30 (1)	KT598043	KT598102
						IV	H31 (4)	KT598043	KT598103
