# Supplementary material for: Unveiling the Identity of Unio crassus (Bivalvia: Unionidae) in Central and Eastern Europe
Source: Genes (Basel). 2026 Oct 14;17(10):1282. doi: 10.3390/genes17101282 (PMC13704050; doi:10.3390/genes17101282)
Supplement: Supplementary file 1 [file genes-17-01282-s001.zip › Table_S2.pdf]

Table S2. DNA polymorphism data for collected individuals of *Unio crassus*.

No.	River (code)	Sample size (N)	Nucleotide diversity (π)*	Haplotype diversity (Hd)*	Number of unique haplotypes (H)	Number of polymorphic sites (S)	Mean number of nucleotide differences (K)	Mean *K2P* genetic distance
1.	Virvyčia (VIR)	3	0.00110	1.000	3	3	2.000	0.001±0.001
2.	Dubissa (DUB)	2	0.00386	1.000	2	7	7.000	0.004±0.001
3.	Zalvė (ZAL)	4	0.00276	0.833	3	9	5.000	0.003±0.001
4.	Babrungas (BAB)	5	0.00232	0.700	3	10	4.200	0.002±0.001
5.	Šešuvis (SES)	3	0.00294	1.000	3	8	5.333	0.003±0.001
6.	Luknelė (LUK)	1	-	-	1	-	-	-
7.	Szeszupa (SZE)	10	0.00262	0.778	6	11	4.756	0.003±0.001
8.	Czarna Hańcza (CZH)	11	0.00317	0.945	9	17	5.745	0.003±0.001
9.	Brzeźniacka Węgorza (BWE)	10	0.00162	0.844	6	12	2.933	0.002±0.000
10.	Słupia (SLP)	10	0.00193	0.644	4	11	3.489	0.002±0.001
11.	Radunia (RAD)	5	0.00089	0.400	2	4	1.600	0.001±0.000
12.	Pilica (PIL)	10	0.01907	0.711	4	67	34.422	0.020±0.003
13.	Warkocz (WAR)	10	0.01232	0.378	3	63	22.244	0.013±0.002
14.	Jasiołka (JAS)	11	0.00070	0.345	3	7	1.273	0.001±0.000
15.	Osławica (OSL)	5	0.00022	0.400	2	1	0.400	0.000±0.000
16.	San (SAN)	10	0.00022	0.378	3	2	0.400	0.000±0.000
17.	Drawa (DRW)	11	0.01561	0.873	7	65	28.182	0.016±0.002
18.	Brzeźniczanka (BRZ)	5	0.00066	0.400	2	3	1.200	0.001±0.000
19.	Chumava (CHU)	11	0.00713	0.818	7	67	12.855	0.007±0.001
20.	Sázava (SAZ)	12	0.00105	0.894	8	9	1.909	0.001±0.000
21.	Jeleśnia (JEL)	7	0.00327	0.524	3	16	5.905	0.003±0.001
22.	Grónik (GRN)	11	0.00375	0.891	8	18	6.764	0.004±0.001
23.	Rába[1] (URA)	13	0.00565	0.974	11	32	10.179	0.006±0.001
24.	Rába[2] (GYO)	4	0.00202	0.833	3	7	3.667	0.002±0.001
25.	Danube (GOD)	1	-	-	1	-	-	-
26.	Ipoly[1] (LET)	6	0.00202	1.000	6	8	3.667	0.002±0.001
27.	Ipoly[2] (IPV)	7	0.00194	0.810	4	8	3.524	0.002±0.001
28.	Ipoly[3] (TES)	5	0.00132	0.900	4	6	2.400	0.001±0.001
29.	Tisza[1] (TIP)	8	0.00309	0.929	6	21	5.607	0.003±0.001
30.	Tisza[2] (TAK)	7	0.00210	0.952	6	12	3.810	0.002±0.001
31.	Tisza[3] (TIB)	5	0.01415	1.000	5	61	25.500	0.014±0.002
32.	Bodrog[1] (BOK)	3	0.00000	0.000	1	0	0.000	0.000±0.000
33.	Bodrog[2] (BOL)	1	-	-	1	-	-	-
34.	Bodrog[3] (FLB)	8	0.00186	0.929	6	12	3.357	0.002±0.001
35.	Talna (TAL)	5	0.00166	0.900	4	7	3.000	0.002±0.001
36.	Siret (SIR)	5	0.00733	1.000	5	30	13.200	0.007±0.001
37.	Bega (BEG)	5	0.00499	0.700	3	19	9.000	0.005±0.001
38.	Ogosta (OGO)	5	0.00521	0.800	3	16	9.400	0.005±0.001
39.	Nishava (NIS)	5	0.00199	1.000	5	8	3.600	0.002±0.001
40.	Palakariya (PAL)	5	0.00000	0.000	1	0	0.000	0.000±0.000
41.	Prut (PRT)	5	0.00322	1.000	5	13	5.800	0.003±0.001
42.	Czerkawa (CZE)	5	0.01530	0.700	3	69	27.600	0.016±0.002
43.	Boh (BOH)	5	0.00022	0.400	2	1	0.400	0.000±0.000
44.	Czorna (CZO)	7	0.00981	0.667	3	37	17.714	0.010±0.002

* for sites with $n < 5$, the indices are reported for completeness but should not be over-interpreted
